# Multi-modal integrated intervention combining exercise, anti-inflammatory, and dietary advice (MMIEAD) for adults with kidney cachexia: protocol for a mixed-methods feasibility cluster randomised controlled trial and process evaluation

**DOI:** 10.1186/s40814-026-01784-z

**Published:** 2026-02-19

**Authors:** Joanne Reid, Carolyn Blair, Adrian Slee, Clare McKeaveney, Alexander P. Maxwell, Vicki Adell, Marion Carson, Faizan Awan, Malcolm Brown, Andrew Davenport, Damian Fogarty, Denis Fouque, Oonagh Gooding, Teresa McKinley, Samantha Hagan, Carolyn Hutchinson, Kamyar Kalantar-Zadeh, Karen Magee, Robert Mullan, Neal Morgan, NICRN Clinical Research Team, Helen Noble, Sam Porter, David S. Seres, Joanne Shields, Ian Swaine, Miles Witham

**Affiliations:** 1 School of Nursing and Midwifery, Queen’s University Belfast, Belfast, UK; 2 Division of Medicine, Faculty of Medical Sciences, University College London, London, UK; 3 Centre for Public Health, Queen’s University Belfast, Belfast, UK; 4 South Eastern Health and Social Care Trust, Renal Unit Ulster Hospital, Upper Newtownards Rd., Belfast, UK; 5 Renal Unit, Antrim Area Hospital, Northern Health & Social Care Trust, Antrim, UK; 6 Renal Patient Led Advisory Network (RPLAN), Lancashire, UK; 7 School of Sport and Exercise Science, Ulster University, Belfast, UK; 8 UCL Department of Renal Medicine, Royal Free Hospital, University College London Medical School, London, UK; 9 Regional Nephrology Unit, Belfast City Hospital, Belfast Health & Social Care Trust, Belfast, UK; 10 Division of Nephrology, Dialysis, and Nutrition, Hôpital Lyon Sud and University of Lyon, Lyon, France; 11 Southern Health and Social Care Trust, Renal Unit, Daisy Hill Hospital, Newry, UK; 12 Irvine Division of Nephrology, Hypertension, and Kidney Transplantation, University of California, Berkeley, USA; 13 NICRN, Clinical Research Team, Belfast Health and Social Care Trust, Belfast, UK; 14 Department of Social Sciences and Social Work, Bournemouth University, Poole, UK; 15 Institute of Human Nutrition and Department of Medicine, Columbia University Irving Medical Center, New York, NY USA; 16 School of Human Sciences, University of Greenwich, London, UK; 17 AGE Research Group, NIHR Newcastle Biomedical Research Centre, Newcastle University, Newcastle upon Tyne, UK

**Keywords:** Cachexia, Multimodal interventions, Haemodialysis, Muscle wasting

## Abstract

**Background:**

Kidney cachexia is a debilitating and under-recognised complication of advanced chronic kidney disease (CKD), characterised by unintentional weight loss, muscle wasting, inflammation, and reduced functional capacity. Its profound impact on morbidity, quality of life, and healthcare utilisation underscores the need for targeted, implementable interventions. The multicomponent implementation strategy for a multi-modal, integrated, exercise, anti-inflammatory, and dietary advice (MMIEAD) intervention seeks to address this gap. Guided by the practical, robust implementation, and sustainability model (PRISM), which incorporates reach, effectiveness, adoption, implementation, and maintenance (RE-AIM) outcomes, this study aims to ensure strong intervention–context alignment to support future scalability.

**Methods:**

The MMIEAD model will be evaluated by determining patient eligibility and recruitment rates, identifying intervention retention and adherence, assessing key statistical and methodological considerations to inform optimal study design and data collection burden, conducting a qualitative process evaluation to examine intervention acceptability and practicality, and determining the feasibility of undertaking a definitive economic evaluation.

**Design:**

This mixed-methods study consists of three phases. Phase 1 will deliver and evaluate a 12-week multimodal intervention using a feasibility cluster randomised controlled trial (cRCT) design. Phase 2 will undertake a qualitative process evaluation with healthcare practitioners (HCPs) and patients. Phase 3 will assess the feasibility of conducting a full economic evaluation.

**Participants:**

Patients will be eligible if they have haemodialysis-dependent CKD stage 5 for more than 3 months, have experienced unintentional weight loss of at least 5% in the previous 12 months, or have a body mass index <20 kg/m^2^, and are aged over 18 years. HCPs will be eligible if they are members of the multidisciplinary healthcare team for more than 3 months and have had exposure to the study.

**Setting and randomisation:**

The study will be conducted across four outpatient haemodialysis units in the UK. Two sites have been randomly allocated to the intervention group and two to the control group.

**Sample:**

For phases 1 and 3, a total of 40 patient participants will be recruited (10 per intervention site and 10 per control site). For phase 2, qualitative data will be collected through interviews with approximately 15 patients and interviews or focus groups with 15 HCPs across all sites. Recruitment commenced on 08.09.25 following ethical approval (REC reference: 25/NI/0069).

**Discussion:**

Using multi-method analyses informed by PRISM/RE-AIM dimensions, we will generate evidence on the feasibility, acceptability, and contextual fit of the MMIEAD intervention to prepare for a definitive UK-wide multi-site cRCT.

**Trial registration:**

Trial registration number NCT07107087 (30 July 2025). https://clinicaltrials.gov/study/NCT07107087

**Supplementary Information:**

The online version contains supplementary material available at 10.1186/s40814-026-01784-z.

## Background

Pathological loss of muscle mass, or kidney cachexia, is a major contributor to morbidity, mortality (20% 1-year mortality rate), increased healthcare costs, and reduced quality of life [1]. Unimodal therapy has limited success in cachectic patient populations, given its multifactorial pathogenesis [2]. This has been recognised in chronic conditions such as cancer which have progressed to the development of clinical guidelines for cancer cachexia multi-modal interventions [3]. Patients with or at risk of cachexia must be supported to maintain body weight, improve strength, enhance the capacity for independent functioning, reduce frailty, stabilise abnormal biochemistry, and prolong survival [4]. Therefore, as systematic reviews and meta-analyses suggest the incorporation of a multi-component intervention, it is essential to address the complex pathophysiology of cachexia [5, 6]. However, currently no such licensed treatment or standard of care for cachexia in kidney failure exists. This is the first study to explore the potential use of a multi-modal, integrated, exercise, anti-inflammatory, and dietary advice (MMIEAD) intervention for kidney cachexia through a feasibility cluster randomised controlled trial (cRCT).

Brownson and colleagues [7] identify three types of evidence in implementation science: type one evidence on aetiology and burden; type two evidence on intervention effectiveness; and type three evidence on dissemination and implementation within context. Progression to a feasibility trial for kidney cachexia reflects more than a decade of evidence generation across all three domains. For type one evidence, our longitudinal pilot study [8] using a generic definition of cachexia in haemodialysis patients reported a 16% prevalence in a UK cohort, confirming kidney cachexia as a severe yet under-recognised condition. Complementary qualitative work including an international mixed-methods study exploring renal HCPs’ perceptions [9] and an interpretive phenomenological study involving patients and carers [10] further demonstrates the substantial burden of cachexia and the need for tailored interventions. Type two evidence has been informed by four reviews of multimodal cachexia interventions [11] which underpin the selection, duration, and structure of MMIEAD. Consistent with scientific consensus, multimodal approaches integrating exercise, nutritional support, and anti-inflammatory strategies are considered the most effective means of treating severe wasting [12]. Evidence suggests that these components act synergistically to improve nutritional and physical status, thereby enhancing quality of life [13]. Given that nutritional interventions alone do not improve longevity [14], active treatment must also target muscle loss [15] and the inflammatory pathways that drive cachexia [16]. Combining pharmacological and non-pharmacological components enables these multifactorial mechanisms to be addressed concurrently [17]. For type three evidence, we employed a theory of change (ToC) framework to strengthen intervention design and clarify the causal pathways underpinning implementation feasibility [18–20]. This work was guided by frameworks such as the Medical Research Council’s guidance on developing and evaluating complex interventions [21], the TIDieR checklist [22], and the SPIRIT 2025 guidelines [23]. Reporting the ToC in advance has enhanced understanding of both contextual influences and the intervention’s active elements [24]. Aligned with updated MRC guidance [25], the intervention theory was developed collaboratively with diverse stakeholders, including patient and public involvement and engagement (PPIE) representatives [20]. The resulting ToC map [20] outlines how core components and mechanisms interact with contextual factors and how these may influence implementation success.

To ensure optimum intervention-context alignment, we have interwoven the Practical, Robust Implementation, and Sustainability Model (PRISM) [26] which incorporates reach, effectiveness, adoption, implementation, and maintenance (RE-AIM) [27] outcomes into our trial design. Considering implementation science emphasises alignment of interventions to local contexts with an equity focus, this underscored a need to understand local contextual domains [28]. PRISM’s context-oriented approach has helped us to prepare accordingly for the intervention, for example, taking into consideration the attributes of those delivering and receiving the intervention and the infrastructure supporting the intervention [26]. We have actively demonstrated an ongoing and iterative engagement process with PPIE representatives through co-design and ongoing involvement in MMIEAD [26]. PPIE representatives’ priorities, needs, and values have been and continue to be deemed equally important alongside organisational perspectives, resources, and infrastructure because these factors impact an intervention’s uptake, equitable implementation, and impact [28]. Our approach has combined both a comprehensive approach that includes clinical, academic, and experiential expertise, as illustrated by our international expert reference group consisting of practicing nephrologists, physiologists, dieticians, clinical research nurses, PPIE representatives, and academics. The core aspects of RE-AIM which we have considered in this feasibility trial include the intervention’s reach, effectiveness, adoption, and implementation [26] as not all RE-AIM objectives can be met within a feasibility study (i.e. maintenance). This framework [26] has been used to inform the planning of research questions, data collection, and outcomes appropriate for a cRCT feasibility study. This approach to designing our feasibility trial will ensure optimum opportunity to address any issues which arise during implementation and evaluation [26].

Aligned with the applied approach of implementation science, our work [29] is directly influencing clinical guidelines through citations such as the European Society for Clinical Nutrition and Metabolism (ESPEN) Guideline on clinical nutrition in hospitalised patients with acute or chronic kidney disease [30]. Our hope is that through this feasibility trial and subsequent definitive trial impact will continue and research findings will be applied into multimodal interventions [31] to help individuals experiencing or at risk of kidney cachexia maintain body weight, and improve strength to enhance their capacity for independent functioning [1]. Using implementation science, we have developed an in-depth understanding of what is needed, how, and why MMIEAD should achieve impact and have designed the components and processes on best evidence available to achieve the strongest external validity possible [32, 33]. The aim of this paper is to describe the study protocol for the cRCT of the MMIEAD intervention.

## Methods/design

### Aim and objectives

#### Primary aim

To determine the feasibility and acceptability of the MMIEAD intervention for adult patients with renal cachexia.

#### Objectives

i. To determine patient recruitment, retention, and adherence rates (to all 3 components of MMIEAD).
ii. To ascertain key considerations for a definitive UK-wide trial including suitability of outcome measures and their potential impacts and data collection burden; optimal study design (including randomisation procedure); and multicomponent intervention design.
iii. To conduct a qualitative process evaluation to assess intervention acceptability and practicality with HCPs and patients.
iv. To assess the feasibility of conducting a definitive economic evaluation of MMIEAD for patients receiving haemodialysis by testing data collection techniques and suitability of outcome measures.

### Study design and frameworks

The MMIEAD study will deliver a 12-week multimodal intervention using a feasibility cRCT design with a process and economic evaluation. Intervention arm (MMIEAD and standard care) vs. control (standard care). This study will use the conceptual framework PRISM [26] and RE-AIM [27] to enable systematic consideration of the strengths and weaknesses of MMIEAD.

PRISM will address context by considering aspects relevant to this feasibility trial:

1. perspectives on the intervention;
2. the external environment;
3. the implementation and infrastructure; and
4. the characteristics of those involved in delivering and receiving an influence programme adoption and implementation

Four of the five dimensions included in the RE-AIM framework apply to the parameters of the proposed study:

1. Reach - describes the number, proportion and representativeness of the patients and HCPs who participate in the research study;
2. Effectiveness – the impact of the intervention on outcomes (given the nature of this trial, we will focus on the feasibility of outcome measures);
3. Adoption – the willingness of participants who initiate the intervention to complete it; and
4. Implementation – fidelity of intervention protocol and consistency of implementation across recruitment sites.

The fifth dimension “maintenance” refers to on-going implementation into routine care for at least 2 years post-intervention and is therefore beyond the scope of this feasibility study.

### Phases of the study

The MMIEAD study has been categorised in three phases as follows:

In phase 1, we will deliver MMIEAD, a 12-week multimodal intervention using a feasibility cRCT design to the intervention sites, and collect outcome measures at three timepoints from both intervention and control sites. In phase 2, we will conduct a process evaluation to assess intervention acceptability and practicality with HCPs and patients, and in phase 3, the feasibility of conducting a definitive economic evaluation of MMIEAD will be assessed.

#### Randomisation

Cluster sites have been randomly placed in either the multimodal and standard care group (intervention group) or the standard care group (control group). Two sites have been randomised to intervention, and two have been allocated to control centrally using a computer-generated randomisation schedule (Nquery) independently by a statistician from Northern Ireland Clinical Trials Unit (NICTU). For pragmatic reasons, the personnel who will approach participants regarding the trial will have access to the random allocation sequence and participants will be made aware of their allocation.

#### Blinding

Due to the nature of the intervention, neither patients nor HCPs can be blinded.

####  Study setting

The research will be conducted in four outpatient haemodialysis sites within the UK. This multi-site approach enhances the diversity of patient demographics and service delivery models represented, strengthening the generalisability and relevance of the feasibility findings.

### Characteristics of participants

For inclusion *criteria and exclusion criteria* for patients, please see Table 1 below.

**Table 1:** Inclusion and exclusion criteria for patients

**Renal population**	
**Inclusion criteria**	**Exclusion criteria**
CKD stage 5 patients receiving maintenance HD therapy for >3 months, have unintentional weight loss of at least 5% in 12 months or BMI less than 20 kg/m^2^, who have self-reported decreased physical function/muscle strength and appetite, increased fatigue, who are male or female, aged >18 years, and able to provide written informed consent.	Patients under 18 years of age
	Patients within 3 months of initiation of HD (patients in this time frame are generally less clinically stable, many having vascular access procedures performed and with much higher rates of intercurrent events, including death and hospitalisation).
	Patients experiencing weight loss due to clinically explainable reasons for example malabsorption or oesophageal blockage.
	Patients already receiving chronic anticoagulation therapy or with a history of bleeding with 3 months/active bleeding issues.
	Patients receiving immunosuppressants or immunomodulators.
	Patients who are pregnant or breast feeding.
	Patients with a hypersensitivity to any of the constituent components of the omega-3 dietary supplement
	Patients who have dementia, a psychiatric disorder (who are not treated and stable) or a severe cognitive impairment which would deem them unable to give informed consent.
	Patients with expected survival on dialysis of <6 months (e.g. those with severe heart failure (New York Heart Association ≥3)).
	Patients for whom dialysis withdrawal is being considered.
	Patients likely to receive a live-donor transplant or transfer to peritoneal dialysis during the study duration.
	Patients with bilateral lower limb amputations.
	Patients unable to walk without aids or assistance.
	Patients deemed to be clinically unstable by their treating physician.
	Patients who are non-English speaking (not having the ability to provide informed consent, read, and write English).
	Patients who are currently enrolled in any study which involves exercise, fish oil/omega-3, or have been taking fish oil or omega-3 supplementation in the previous 3 months.
	Are not able/willing to be involved.

For inclusion and exclusion criteria for HCPs, see Table 2 below.

**Table 2:** Inclusion and exclusion criteria for HCPs

**Healthcare professionals**	
**Inclusion criteria**	**Exclusion criteria**
18 years of age and over	Informal carers
Currently working within the intervention or control sites as a healthcare professional for three months or over.	
Caring for a patient enrolled in the study with end-stage kidney disease and in receipt of haemodialysis.	No involvement in or exposure to the study.
Can provide informed consent, read and write in English.	
Able/willing to be involved.	

### Description of the intervention

#### The components of MMIEAD are as follows

##### Intervention arm (MMIEAD and standard care)

In addition to their standard haemodialysis care, the intervention group will receive MMIEAD, an individualised exercise programme, oral omega-3 supplementation, and dietary advice. All study sites have been surveyed to ensure the intervention components are not provided in standard care, e.g. combined specialist dietetic support with individualised exercise programmes in haemodialysis management.

##### Individualised exercise programme

Mechanical tension induced by aerobic and resistance exercise can increase the rate of metabolic stress and stimulate subcellular pathways involved in muscle protein synthesis which may play a role in exercise-induced muscle growth [34]. Aligned with PRISM [26], exercise components have been co-designed by patients, physiotherapists, and expertise within the research team and are based on the EXACT exercise intervention for patients with advanced cancer [35]. Each participant will receive an exercise handbook and exercise diary to complete daily consisting of 12-week home-based progressive, moderate-intensity walking, and resistance exercise, two–five times per week. Participants will have the flexibility to complete walking and resistance exercises consecutively or separately based on readiness (e.g. symptom burden) or preference, and modifications will be made. Moderate-intensity walking has been defined in the handbook and will be explained to participants using simple, standardised guidance based on the Borg Rating of Perceived Exertion (RPE) scale and functional descriptors. Specifically, participants will be advised that moderate intensity corresponds to an RPE of 12–13 (“somewhat hard”) and should feel like a pace at which they are breathing faster but can still maintain a conversation. This autoregulated programme will involve muscle strengthening and balance retraining exercises, progressing in difficulty over the 12 weeks. Autoregulation refers to the permission of dose modifications to reduce the intensity of exercise and catch up on missed exercise based on individual readiness and has been pivotal in delivering exercise for patients where the disease burden is complex [35]. The exercise programme was informed by the EXACT trial [35], including the use of body mass exercises, dumbbells, and household items to ensure accessibility. During the co-design process with patients, carers, and multidisciplinary stakeholders, modifications were introduced to enhance acceptability and feasibility for adults receiving haemodialysis with cachexia. These refinements included tailoring exercise options to accommodate the functional capacity of those receiving HD. The final programme comprises a structured core set of exercises adapted from EXACT, with individualisation based on the physiotherapist/suitably qualified member of the research team’s initial assessment and weekly reviews. The intervention incorporates established behaviour change techniques (BCTs) to support engagement with the exercise intervention, including goal setting, action planning, self-monitoring, problem-solving, and feedback on behaviour. These techniques are embedded within patient-facing booklets to promote adherence to the exercise sessions programme. Patients will be reviewed by the physiotherapist at the start of the intervention and at 6 weeks to assess their ability to engage in the exercise component and competency to progress.

##### Dietary advice

Dietary advice focuses on adequate dietary protein intake to promote systemic protein anabolic effects required to treat severe muscle wasting [36]. Each patient in the intervention group will receive a 60-min personalised dietetic assessment. Specific to this study, this dietetic programme will focus on adequate dietary protein and energy intake to promote systemic protein anabolic effects required to treat severe muscle wasting (e.g. Kidney Disease Outcomes Quality Initiative (KDOQI) Clinical Practice Guideline For Nutrition in Chronic Kidney Disease: 2020 Update: dietary protein intake of 1.0–1.2g/kg 35 kcal/kg per day adjusted to ideal body weight and standardised across intervention sites) [37]. In addition, this programme will take account of patients’ co-morbidities in relation to dietary advice provided. Patients will be reviewed by the dietician at the start of the intervention and at 6 weeks. Patients will also complete a 3-day food diary on 5 occasions throughout the 12 weeks of the interventions (weeks 1, 3, 6, 9, 12).

##### Oral omega-3 fatty acid supplement

Targeting inflammatory pathways provides a window of opportunity for parallel interventions (e.g. exercise) to take effect. Nutritional supplements with anti-inflammatory effects, e.g. omega-3 fatty acids, are associated with reduced chronic inflammation in patients with kidney failure and could inhibit the inflammatory cytokine pathways associated with cachexia [38].

Omega-3 fatty acids are considered a safe and potentially therapeutic treatment for patients at high risk of inflammation such as those with kidney cachexia. Each patient will receive a 12-week supply of omega-3 fatty acid oral capsules using a daily dose (1000mg natural triglycerides, eicosapentaenoic acid (EPA) 180 mg, docosahexaenoic acid (DHA) 120 mg twice daily) which has a proven safety profile, tolerability, reduces inflammatory markers, and attenuates muscle protein breakdown in haemodialysis patients [39]. The capsules will be given to patients during the initial home visit at the commencement of the study.

##### Weekly contact

Patients in the intervention arm, will be contacted weekly (or more if preferred) by a telephone. A log will be completed regarding their adherence to the exercise, dietary advice, and nutritional supplementation. Weekly contact will be carried out by a trained member of the research team. This contact will provide behavioural support, monitor adherence, address barriers, and reinforce the BCTs integrated into the intervention.

#### Control arm (standard care)

Patients in the control arm will not be offered the MMIEAD intervention and will receive standard care alone. The control arm will complete the assessments (see the ‘outcomes’ section below) at the three timepoints (baseline, 6, and 12 weeks). Patients who have been allocated to the control group will continue to receive routine standard care throughout the study. As the recruitment sites cover four hospital sites, a description of standard care per recruitment site will be collected as part of the process evaluation.

#### Processes

##### Recruitment (phase 1 and 3)

Our recruitment rate is anticipated to be two patients per month per site. The sample size for the feasibility study is 40 patients, collectively 20 per intervention arm, 20 per control arm, based on providing sufficient information to inform the primary outcomes [40]. The study is being supported by Northern Ireland Clinical Research Network (NICRN).

##### Recruitment for the process evaluation (phase 2)

Approximately 15 patients will be recruited into the process evaluation. Approximately five patients from control, five intervention completers (across both intervention sites), and five intervention non-completers (across both intervention sites).

Approximately 15 healthcare staff across all sites will be recruited into either focus groups or individual interviews, with a proposed total of three focus groups. The focus groups will be limited by the number of staff available who meet the eligibility criteria and the requirement to produce a manageable amount of data for the time limitations of the study.

**Fig. 1:**
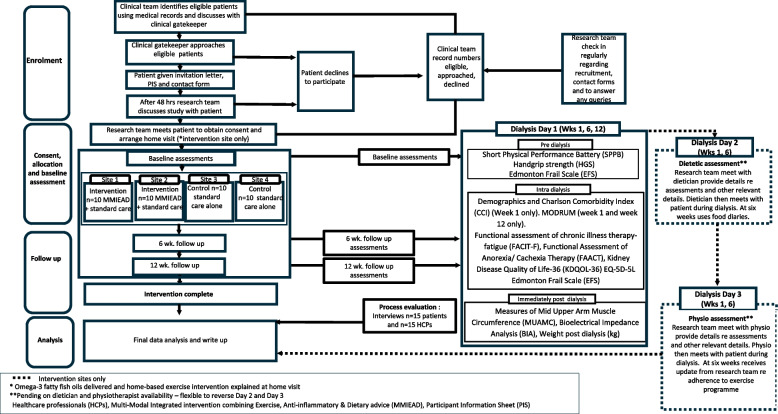
Study flow diagram

Data saturation will be reached when no new themes are found within the data [41] (see Fig. 1).

### Data collection

To ensure consistency across the four study sites, all outcome assessors have undertaken standardised training before data collection begins, with refresher training provided as required. Harmonised data collection protocols, standard operating procedures, and uniform measurement tools will be used across all locations. Regular cross-site monitoring and fidelity checks will be conducted to ensure adherence to procedures and to minimise measurement error.

#### Phase 1

All eligible patients in both intervention arms and control arm will be required to complete baseline and follow up data at 6 weeks and 12 weeks. Longer-term survival (at 6 months and 12 months from the date of recruitment) will also be recorded. Adverse events (AEs) and serious adverse events (SAEs) will be reported using a standard procedure and relatedness will be determined by the clinical team as per our ethically approved reporting structure.

##### Primary outcome

Recruitment is measured as the number of fully enrolled patients as a proportion of approached eligible individuals. The appropriateness of this outcome in a feasibility study is highlighted in the literature [42]. Retention will be assessed by the proportion of patients screened vs. those consented and attrition rates (at 6 weeks and 12 weeks).

In order to test data collection techniques and suitability of outcome measures, secondary outcomes include demographic data (including age, height to calculate BMI), dialysis vintage, and co-morbidities (Charlson comorbidity index (CII)) [43]; these will be collected at baseline only. The following outcomes will be collected from all patients prior to commencement (baseline), midway (6 weeks), and on completion (12 weeks) of the intervention:

##### Physical function

The Short Physical Performance Battery (SPPB) [44] will provide co-efficacy endpoints of both muscle strength and functionality which are clinically meaningful outcome measures for sarcopenia and frailty and well established in haemodialysis patient populations. More specifically, the Edmonton Frail Scale (EFS) [45] will be included which is validated for use in haemodialysis population.

##### Body weight/weight loss

Unintentional weight loss by body weight (kg) monitoring will be recorded [46].

##### Muscle mass

Three methods of muscle mass will be used including bioelectrical impedance analysis (BIA) using a calibrated dual-frequency (5 and 50 kHz) Bodystat 1500 MDD device (Bodystat, Isle of Man, UK). A standard protocol will be followed with BIA assessment post-dialysis to control for variation in fluid status [47]. Measurements will be taken in the supine position, with electrodes attached on the hand and foot contralateral to the side of the arteriovenous fistula, and at constant room temperature. Patients with any implantable electronic devices (such as pacemakers) will be excluded from this assessment as per the manufacturer’s guidelines. For those patients who complete the BIA, a measure of BMI will be calculated using the clinical formula weight (kg)/height (m^2^). Mid-arm circumference (MAC) and triceps skinfold (TSF) thickness (TSF in triplicate and the average calculated) will be measured in the non-fistula arm using a tape measure and Harpenden skinfold calliper set respectively. The mid upper arm muscle circumference (MUAMC) will be calculated using the formula MAMC (cm) = MAC (cm) - 0.314 × TSF (mm). Suitable cut-point values designated the 5th percentile as an appropriate cut-point for low MUAMC using normative values (i.e. <23.8 cm for males and <18.4 cm for females) [48, 49] which will provide reliable and valid assessment of muscle mass in the absence of gold standard techniques (e.g. DEXA, MRI or CT). Combining these assessments reduces measurement error and normative values are well established in haemodialysis patient populations [50].

##### Muscle strength

Handgrip strength (HGS) will be measured using a KERN MAP 80K1S hand grip dynamometer which is an ideal device to determine reduced hand strength. A hand grip dynamometer is a highly reliable measurement tool in patient populations and established as a valid tool in haemodialysis populations [47]. We will be using a standard protocol (dominant arm, seated position with elbow at 90°, allowing three attempts) [51]. Specific cut-off points will be applied based on the European Working Group for Sarcopenia in Older People (EWGSOP) for muscle strength (<27 kg for males and <16 kg for females) [52].

##### Fatigue

The Functional Assessment of Chronic Illness Therapy (FACIT-F) [53] has been used extensively in kidney disease. Brown et al. [54] reported strong correlations between the “chair-rise” time test and FACIT, suggesting that it is a reliable measure of physical function. Lower scores of the FACIT subscale refer to lower function and thus increased fatigue. The optimal cut-off value for FACIT is <30 [55].

##### Appetite

Appetite will be recorded using the Functional Assessment of Anorexia/Cachexia Therapy (FAACT) tool [56]. The optimal cut-off value for the FAACT is <32 [57]; lower scores on FAACT reflect poor appetite. This tool has been used extensively in patient populations and is regarded as a reliable and valid tool in haemodialysis patients and suitable for measuring appetite in patients with cachexia [47].

##### Routine blood values and biomarkers

These include blood values and biomarkers from routine collected bloods including the following: C-reactive protein (CRP), serum albumin, urea reduction ratio (URR), estimated glomerular filtration rate (eGFR), and haemoglobin.

##### Quality of life

Health-related quality of life (HRQOL) has become increasingly important as an outcome measure of kidney replacement therapy, peritoneal dialysis, and transplant. HRQOL among haemodialysis patients has been shown to be lower as compared to pre-dialysis populations, the general population, and other chronic disease populations such as congestive heart failure, diabetes, depression, and even cancer. HRQOL is also poorer in cachectic patient populations and therefore an important criterion [10]. The 36-item Kidney Disease Quality of Life scale (KDQoL-36) will also be administered to patients in conjunction with EQ-5D-5L at baseline, post-intervention, and at 6 weeks and 3 months follow-up (this will be used in conjunction with Modular resource Use Measure (MODRUM), see phase 3). The EQ-5D-5L is recommended by the National Institute of Clinical Excellence (NICE) for deriving utility values for the calculation of quality adjusted life years in cost-effectiveness analysis within definitive economic evaluations [58] and has been shown to be a valid measurement of health status in patients receiving haemodialysis [59].

##### Mortality

Survival will be recorded 6 and 12 months from the date of recruitment, and data will be generated from patients’ medical notes.

#### Phase 2

The process evaluation will follow the MRC guidance on process evaluations for complex evaluations [60] informed by the PRISM/RE-AIM framework [26]. It will therefore focus on the acceptability of the intervention, barriers and facilitators to implementation, the appropriateness of outcome measures, motivations for participation, reasons for continued engagement, and potential mechanism of impact of the intervention. The interviews will be conducted in person, and the guides will be flexible to ensure that participants can express and explore perspectives that they consider relevant to implementation. The interviews will be used to examine the fidelity and quality of implementation, and how the intervention effects are mediated by different settings and contexts. It will also examine acceptability and unintended consequences.

#### Phase 3

Healthcare resource use of patients will be collected using MODRUM [61]. This measure will collect information on health and social care service use pre and after the intervention. The measure will be used at baseline and 3 months follow-up. The outcome of interest will be the feasibility of using a healthcare resource use log in a full economic evaluation according to proportion of patients who complete the healthcare resource use log. The evaluation of costs related to the intervention and implementation, in addition to any operational costs, will provide important information on adoption and implementation in RE-AIM [27].

### Ethics and rigour

This study has been approved by National Health Service (NHS)/Health and Social Care (HSC) Research Ethics Committee (REC reference: 25/NI/0069). To ensure informed consent is obtained freely from participants (both patients and HCPs), they will initially be approached by the local collaborator on site, who will provide them with a verbal summary of the project and the participant information sheet (PIS). Essential aspects of good practice, including clear and sensitively written participant facing materials’ voluntary participation, and informed consent will be employed as a minimum standard. All data will be stored for a minimum of 5 years and subsequently destroyed in accordance with the data protection act [62]. The trial will be conducted in accordance with the protocol as registered on clinical trials (NCT07107087) the principles of the Declaration of Helsinki and Good Clinical Practice (GCP). Medicines and Healthcare Products Regulatory Agency (MHRA) have agreed that the supplement (omega-3 fatty acids) is not a Clinical Trial of an Investigational Medicinal Product (IMP) as defined by the EU Directive 2001/20/EC. The end of the study will be defined as the date of the last visit of the last participant or the completion of any follow-up monitoring and data collection as described in the “Data collection” section.

### PPIE involvement

PPIE representatives have been part of the programme of kidney cachexia work from inception (2015) and have been involved in the design of the study, including the development of a lay abstract (not presented within this paper) and the intervention components. Two representatives (WJ and FA) are part of our expert reference group for MMIEAD and contribute to the development and review of patient-facing materials. Aligned with PRISM guidance [26], we have engaged participants in the co-creation of the intervention and implementation strategies to fit local context and enhance equity.

### Analyses

#### Quantitative analysis (phase 1)

Quantitative data will be coded and entered into the Statistical Package for the Social Sciences (IBM SPSS Statistics Version 24). Data on participant eligibility, recruitment, refusal, and retention will be assessed as proportions and rates. Demographic characteristics and adherence to the intervention components will be reported with descriptive statistics as appropriate, for instance, reporting of mean standard deviation (SD) for interval scale variables and frequency/percentages for categorical variables. Differences between control and intervention groups at baseline will be assessed using chi-squared tests and independent *t*-tests. Potential effective outcomes will estimate the effect size and be analysed for mean change using pair sample *t*-tests to compare the difference pre and post intervention, and repeated measure analysis of variance (ANOVA) to consider interactions. However, given that this is a feasibility study, no inferences will be made regarding statistical significance, given that the sample size is not powered to make such assertions. Data from the 3-day food diary will be entered into Nutritics for diet analysis to estimate dietary protein and energy intake. By using Nutritics software (www.nutritics.com - version 3.1), this will enable analysis of compliance with food-based dietary guidelines [63].

#### Qualitative analysis (phase 2)

The semi-structured interviews and/or focus groups will be recorded and transcribed verbatim. Inductive thematic analysis will be used to analyse the data collected. Thematic analysis will involve identifying and coding central themes within qualitative data through an iterative process [64]. To develop an understanding of the intervention, trial, context, and mechanisms of change, data analysis will be focused on answering these research questions [65]. Themes will be identified at a semantic level, in that they will develop from the explicit content contained within the data. This is different to identifying themes at a latent level, which requires a more hermeneutic approach [64]. Semantic themes are appropriate for a process evaluation as the aim is to gather explicit information on the implementation of an intervention and trial processes [66]. The importance of objective oversight has been highlighted in the MRC guidance on process evaluations, due to potential bias that researchers involved in the development of an intervention may bring when evaluating its acceptability [60]. In order to minimise risk of bias, investigator, method, and data source triangulation will be used to ensure validity of the identified themes [67]. Data will be managed using NVIVO 15.

#### Quantitative analysis (phase 3)

A cost-consequence analysis is recommended by the National Institute of Health Research for both complex interventions and feasibility studies where the outcomes of interest are not clear [68]. Costs and outcome measure data for both the intervention and control group will be presented using means and 95% confidence intervals to show a general overview of the economic costs of the intervention, and differences in resource use and outcome measures between groups. Completion rates and missing data for MODRUM [61] and the EQ-5D-5L [58] will be presented as frequencies to assess the feasibility of data collection.

#### Multi-method integration

Table 3 displays the RE-AIM [27] dimensions and concepts, source materials, instrument or measurement, and the PRISM [26] dimensions and concepts. As needed, the research team will refer to coded transcript data and quantitative findings to further contextualise results. Using the collective evidence will explore the facilitators and barriers at intervention and control sites. We will rate the influence on each RE-AIM [27] outcome as “positive”, “negative”, or “mixed” (i.e. both positive and negative evidence); we will then compare these across intervention and control. These data will inform progress to and potentially the development of the definitive trial.

**Table 3:** PRISM/RE-AIM dimensions and concepts

**RE-AIM dimension and concepts**	**Source material**	**Instrument or measurement**	**PRISM dimension and concepts**
**Reach**			
Inclusion/exclusion criteria	Screening log, HCP interviews; metrics	% screened; % approached; % declined (reasons)	Recipient characteristics and perspectives: Was MMIEAD deemed relevant, accessible, and culturally acceptable by the patients who participated? (intervention only) External environment: Did the external environment (recruitment efforts, advertisement through poster, PIS) facilitate engagement in the trial?
Individuals who enrolled in MMIEAD (intervention sites only)	Collected by the research team	% enrolled. % dropped out	
Characteristics of patients enrolled in trial	Demographic profile, medical records, and CCI	Age, sex, race/ethnicity, marital status, complexity (e.g., comorbidities)	
Patient recruitment	Screening log	Steps utilised by clinical teams to recruit/reach patients	
**Effectiveness**			
Overall feasibility	Adherence data – exercise diaries, 3-day food diaries, tablet count (intervention sites only) Weekly log (from phone calls) Outcome measures Record of AEs and SAEs Patient interviews	% of patients who completed exercise diaries and 3-day food diaries. Nutritics for diet analysis, assess adherence to dietetic advice % of patients who consumed recommended intake of omega-3 fatty fish oil. Identify reasons why adherence potentially affected % outcome measures completed % of related AEs/SAEs Burden of outcome measures	Intervention characteristics: Was MMIEAD adaptable to each individual patient and does it indicate that it is beneficial without causing any harm? (intervention only) Recipient experience: Did patients’ perceptions indicate that the MMIEAD intervention could improve QoL? (intervention only)
Variations in feasibility	HCP interviews	Identify barriers, facilitators, potential reasons for variations in feasibility.	
Patients’ satisfaction with the intervention (intervention sites only)	Patient interviews	Questions on acceptability of trial organisation, physical activity dietary advice, nutritional supplement weekly support.	
**Adoption**			
Level of staff adoption (intervention sites only)	HCP interview	Staff adoption of the MMIEAD intervention - awareness, and receptivity.	Organisational characteristics and infrastructure: Did clinical team deem it a feasible intervention? Were there adequate resources, staff training, and support? External environment: Did specified time allocation for CRN, dietetic, physio encourage uptake, or is more needed?
Patient drop out	HCP interview	% patients dropped out of the trial across intervention and control, reasons.	
Patient participation in MMIEAD (intervention sites only)	Patient interviews	Completers and non-completers issues/challenges, encountered with MMIEAD or personal health.	
**Implementation**			
Adherence to intervention guidelines (intervention sites only)	HCP and patient interviews; metrics from exercise diaries, food diaries, and tablet count	% of patients who completed exercise programme, dietary advice and complied with recommendations for omega-3 supplementation	Implementation infrastructure: Was there adequate feedback, and support for the clinical team from the research team to maintain fidelity? Adaptation support: Did MMIEAD allow for tailoring without undermining core elements?
Adaptations made	HCP interviews	Identify adaptations made, reasons why.	
Feasibility of economic measure	MODRUM, EQ-5D-5L	Feasibility of using these measures for health and social care service use and deriving utility values for the calculation of quality adjusted life years	

*AEs* adverse events, *CCI* Charlson comorbidity index, *CRN* clinical research nurse, *HCPs* healthcare professionals, *MODRUM* Modular resource Use Measure, *MMIEAD* Multi-Modal Integrated intervention combining Exercise, Anti-inflammatory, & Dietary advice, *PIS* participant information sheet, *QoL* quality of life, *SAEs* serious adverse events

#### Missing data

As this is a feasibility study, the primary aim regarding missing data is to assess its extent, patterns, and potential sources to inform procedures for a future definitive trial. Missing quantitative data will not be imputed; instead, descriptive analyses and exploratory sensitivity checks will be conducted to understand the impact of missingness. For qualitative data, interviews or focus groups that are incomplete or not recorded due to participant withdrawal will not be replaced, and analysis will proceed using available transcripts. All missing data will be documented and reported transparently.

## Discussion

This is the first study to explore the potential use of a MMIEAD for kidney cachexia; therefore, a feasibility study is appropriate to test (and potentially refine) the intervention components and their efficacy, while addressing the relevant questions surrounding feasibility [42]. Sample size for quantitative and qualitative analyses is often contended in feasibility studies as are the parameters around progression to a definitive RCT. Adherence to Pearson and colleagues [69], guidance regarding aims, methods, design, measures appropriate for those undertaking formative feasibility, or pilot studies in the field of implementation science has been previously described in the “Methods/design” section of this paper. In this discussion, we will describe the rationale for the sample size and our planned progression criteria to assess implementation feasibility as per Person and colleagues [69] guidance.

In quantitative analyses, there is little consensus on the appropriate sample size for a feasibility study, with guidance ranging from 12 per arm [70] to 50 per arm [71]. The justification for larger sample sizes in feasibility studies is often based around obtaining narrow standard deviations on outcome measures to maximise precision in a future power calculation [71]. However, there is disagreement over whether this is an appropriate method to inform power calculations as this would in turn increase the likelihood of identifying a statistically significant effect during the feasibility stage, which can be inappropriately reported, call into question the validity of the effect, and reduce the likelihood of a follow-up RCT [72–74]. As per the CONSORT extension [75], feasibility studies should focus on the acceptability of trial processes or an intervention, including recruitment, the time needed to collect and analyse data, and response rates to outcome measures. The overall evaluation of MMIEAD will require a multi-methods approach, with both quantitative and qualitative data. In qualitative analyses, for the process evaluation, simulations have shown that a sample size of 10 should identify 80% of problems within a complex intervention [76] while data saturation is likely to be reached at 12 interviews on average [41]. The principle of 10 plus five for data saturation outlines that a minimum of 10 interviews should be conducted, followed by potentially 5 consecutive interviews that present no new findings [77]. A formal sample size calculation is not appropriate for feasibility studies as the objectives do not include establishing the effectiveness of an intervention.

Progression to a definitive RCT will be determined by recruitment rates and the acceptability of the intervention for patients and staff. When developing progression criteria, the use of guidelines is suggested, rather than using strict thresholds, to allow for appropriate interpretation and exploration of potential solutions [75]. For example, Pearson and colleagues [69] recommend the use of a traffic light system with varying levels of acceptability. Therefore, compliance with the multimodal intervention will be assessed according to individual components (e.g. number of exercise sessions/tablet count) and thresholds of <50%, 50–80%, and >80%. Compliance of ≥50% of the specific intervention in ≥50% of patients will be considered acceptable; the appropriateness of which is outlined in previous multimodal interventions in cachexia management [78]. Progression to a full trial will be contingent on the acceptability of the intervention for both patients and staff regardless of recruitment rates which will be assessed within the qualitative process evaluation [66]. Any necessary modifications identified within the process evaluation to improve the intervention will be made prior to progression to a definitive trial [76].

### Strengths and limitations

The multi-phase study design, guided by the RE-AIM-PRISM framework [26, 27], will generate evidence of feasibility and implementation outcomes to build a strong definitive RCT and further refine our ToC model [20]. However, complex interventions contain several interacting components and present practical and methodological difficulties, such as standardising its design across two study sites, and adapting it to ensure it does not negatively impact on standard care [79]. Through our PRISM/RE-AIM design of MMIEAD, we have been circumspect in preparing for potential contextual implementation challenges [26]. The potential barriers and/or logistical challenges are described as follows, together with the mitigation strategies for overcoming them.

1. We are aware that standard care may differ at site level; therefore, we plan to record these differences through the process evaluation.
2. The on-site research team is small consisting of three staff with placement agreements for each site; one member of the research team will be responsible for intervention delivery, and therefore to enhance validity, this researcher will not be involved in interviewing participants from the intervention sites for the process evaluation.
3. To ensure consistency in measurements, all on-site research team members have collectively conducted training in physical assessments (BIA, HGS, EFS, SPPB, MUAMC, TSF).
4. We understand that the HCPs involved may experience staffing difficulties and unprecedented pressure; therefore, we have designed the trial to minimise unnecessary burden and support has been ongoing and delivered during online and in-person study set-up meetings.
5. To ensure all member of our wider expert reference group stay informed about study progress, we have scheduled regular meetings and monthly newsletters with reminders and information related to the intervention, to ensure all members are kept updated.
6. Patients receiving haemodialysis with or at risk of kidney cachexia are likely to be subject to multiple physiological challenges as their condition deteriorates which may affect adherence to MMIEAD. In planning this study, we have worked closely with consultant nephrologists who are our local collaborators to ensure that data collection points will coincide with planned dialysis, and routine clinical data already available (such as monthly blood tests performed in all dialysis patients as part of their standard care) will be used to minimise any potential additional burden to patients. Furthermore, PPIE representatives have provided expert patient knowledge and experience from inception taking account of any potential patient burden.

Overall, our preparation for these possible challenges will be ascertained during the process evaluation and their impact on the external validity of the study assessed through our multi-methods analyses using PRISM/RE-AIM dimensions and concepts.

## Supplementary Information

Supplementary Material 1.

Supplementary Material 2.

## Data Availability

All information is included in the manuscript and supplementary file 1.

## Abbreviations

KDQoL-36: 36-item Kidney Disease Quality of Life scale
AEs: Adverse events
ANOVA: Analysis of variance
BIA: Bioelectrical impedance analysis
BMA: Body mass index
CCI: Charlson comorbidity index
CKD: Chronic kidney disease
CRN: Clinical research nurse
cRCT: Cluster randomised controlled trial
CRP: C-reactive protein
DHA: Docosahexaenoic acid
EFS: Edmonton Frail Scale
EPA: Eicosapentaenoic acid
eGFR: Estimated glomerular filtration rate
ESPEN: European Society for Clinical Nutrition and Metabolism
EWGSOP: European Working Group for Sarcopenia in Older People
FACIT-F: Functional Assessment of Chronic Illness Therapy
GCP: Good clinical practice
HGS: Handgrip strength
HRQOL: Health-related quality of life
HCPs: Healthcare professionals
KDOQI: Kidney Disease Outcomes Quality Initiative
MUAMC: Measures of mid upper arm muscle circumference
MRC: Medical Research Council
MUAMC: Mid upper arm muscle circumference
MAC: Mid-arm circumference
MODRUM: Modular resource use measure
MMIEAD: Multi-modal integrated intervention combining exercise, anti-inflammatory, & dietary advice
HSC: National Health Service (NHS)/Health and Social Care
NICE: National Institute of Clinical Excellence
NICTU: Northern Ireland Clinical Trials Unit
NICRN: Northern Ireland Clinical Research Network
PIS: Participant information sheet
PRISM: Practical, robust implementation, and sustainability model
QoL: Quality of life
RCT: Randomised controlled trial
RE-AIM: Reach, effectiveness, adoption, implementation, and maintenance
REC: Research Ethics Committee
SAEs: Serious adverse events
SPPB: Short Physical Performance Battery
SD: Standard deviation
SPSS: Statistical Package for the Social Sciences
TIDieR: Template for intervention description and replication
FAACT: The functional assessment of anorexia/cachexia therapy
ToC: Theory of change
TSF: Triceps skinfold
URR: Urea reduction ratio
FDA: US Food and Drug Administration
